# Phytochemicals inhibit migration of triple negative breast cancer cells by targeting kinase signaling

**DOI:** 10.1186/s12885-019-6479-2

**Published:** 2020-01-02

**Authors:** Pradip Shahi Thakuri, Megha Gupta, Sunil Singh, Ramila Joshi, Eric Glasgow, Alexander Lekan, Seema Agarwal, Gary D. Luker, Hossein Tavana

**Affiliations:** 1 Department of Biomedical Engineering, The University of Akron, Akron, OH 44325 USA; 2 Department of Arts and Sciences, The University of Akron, Akron, OH 44325 USA; 3 Lombardi Cancer Center, Georgetown University Medical Center, Washington, DC 20007 USA; 4 Department of Pathology, Center for Cell Reprogramming, Georgetown University Medical Center, Washington, DC 20007 USA; 5 Department of Radiology, University of Michigan, Ann Arbor, Michigan 48109 USA; 6 Department of Microbiology and Immunology, University of Michigan, Ann Arbor, Michigan 48109 USA; 7 Department of Biomedical Engineering, University of Michigan, Ann Arbor, MI 48109 USA

**Keywords:** Phytochemical, TNBC, Cell migration, Invasion, Metastasis, Protein kinases

## Abstract

**Background:**

Cell migration and invasion are essential processes for metastatic dissemination of cancer cells. Significant progress has been made in developing new therapies against oncogenic signaling to eliminate cancer cells and shrink tumors. However, inherent heterogeneity and treatment-induced adaptation to drugs commonly enable subsets of cancer cells to survive therapy. In addition to local recurrence, these cells escape a primary tumor and migrate through the stroma to access the circulation and metastasize to different organs, leading to an incurable disease. As such, therapeutics that block migration and invasion of cancer cells may inhibit or reduce metastasis and significantly improve cancer therapy. This is particularly more important for cancers, such as triple negative breast cancer, that currently lack targeted drugs.

**Methods:**

We used cell migration, 3D invasion, zebrafish metastasis model, and phosphorylation analysis of 43 protein kinases in nine triple negative breast cancer (TNBC) cell lines to study effects of fisetin and quercetin on inhibition of TNBC cell migration, invasion, and metastasis.

**Results:**

Fisetin and quercetin were highly effective against migration of all nine TNBC cell lines with up to 76 and 74% inhibitory effects, respectively. In addition, treatments significantly reduced 3D invasion of highly motile TNBC cells from spheroids into a collagen matrix and their metastasis in vivo. Fisetin and quercetin commonly targeted different components and substrates of the oncogenic PI3K/AKT pathway and significantly reduced their activities. Additionally, both compounds disrupted activities of several protein kinases in MAPK and STAT pathways. We used molecular inhibitors specific to these signaling proteins to establish the migration-inhibitory role of the two phytochemicals against TNBC cells.

**Conclusions:**

We established that fisetin and quercetin potently inhibit migration of metastatic TNBC cells by interfering with activities of oncogenic protein kinases in multiple pathways.

## Background

Cell migration is essential for progression of cancers to incurable metastatic disease [1, 2]. In a primary tumor environment, cancer cells migrate away from the tumor mass to access blood or lymphatic circulation and intravasate. Additionally, following extravasation in distant organs, cancer cells migrate into the stroma to colonize and form metastases. Both histopathological examinations of human tumors and intravital imaging studies in mice show that cancer cells may migrate individually, as loosely-attached cell streams, and collectively while maintaining cell-cell adhesions [3]. During migration, cancer cells undergo dramatic morphological and structural changes such as cytoskeletal reorganization to develop membrane protrusions [4], and rupture and repair of the nuclear envelope to facilitate movements through confined spaces [5]. Both cell intrinsic factors and tumor microenvironments regulate the migration mode [6, 7]. Despite potential benefits of blocking cancer cell migration to prevent metastasis, the majority of existing therapeutics are only designed to kill proliferating cancer cells or suppress active oncogenic signaling. Although these therapies show some initial success against tumor growth, they have limited or no efficacy against metastasis.

Identifying chemical compounds with the potential to inhibit metastatic cell migration is critically important for cancers with high prevalence of metastasis and without molecular therapy options, such as triple negative breast cancer (TNBC). Despite comprising only ~ 15% of all breast cancers, TNBC is an aggressive disease with poorer prognosis than other subtypes of breast cancer [8]. Due to the lack of estrogen and progesterone receptors and low HER2 expression on TNBC cells, patients do not have targeted therapy options available with other breast cancer subtypes. Molecular profiling of TNBC tumors and understanding of the disease drivers have recently led to some novel discoveries for TNBC [9]. It was shown that tubulointerstitial nephritis antigen-like 1 suppresses TNBC progression and inhibits metastasis in mice by simultaneously blocking fibronectin-integrin binding and EGFR dimerization, thereby inhibiting downstream oncogenic kinase pathways that drive growth and metastatic migration of cancer cells [10]. Inhibition of actin-bundling activity of fascin, which facilitates membrane protrusions and cell motility, by a natural compound analog significantly blocked migration and metastatic colonization of the TNBC cells [11]. Disrupting formation of membrane protrusions and actin polymerization by targeting an actin-related protein complex subunit using benproperine blocked migration of TNBC cells in vitro and metastasis in mice [12]. These promising studies demonstrated that inhibition of cell migration may be used as a therapeutic strategy against cancer metastasis and highlighted the need for novel anti-metastatic compounds.

To address this need, we recently screened 20 natural compounds against migration of two metastatic TNBC cell lines and quantitatively ranked their effectiveness [13]. From this collection, fisetin and quercetin most effectively reduced migration of TNBC cells dose-dependently and at sub-lethal concentrations. Our preliminary analysis showed that fisetin and quercetin significantly reduced activity of MAPK pathway. Additionally, the treatments scavenged intracellular reactive oxygen species that regulate cell motility through activation of kinase signaling [14]. To evaluate to what extent these two phytochemicals have broad activities against TNBC cell migration and to understand the underlying molecular mechanisms, here we screened these compounds against a panel of TNBC cells and performed a comprehensive molecular analysis of oncogenic protein kinases implicated in cell migration. We demonstrated that fisetin and quercetin effectively reduced migration and matrix invasion of TNBC cells in vitro and TNBC cell metastasis in zebrafish. Our comprehensive molecular analysis showed a significant, broad inhibition of oncogenic kinases in TNBC. While these phytochemicals do not show the specificity that molecular inhibitors have against a single target, their activity against multiple signaling molecules offers the advantage of targeting several pathways without having to use combinations of several inhibitors that often cause excessive toxicity [15, 16]. Overall, this study established the potential of fisetin and quercetin as therapeutic agents against metastatic TNBC and identified multiple molecular targets that can be harnessed for TNBC treatment.

## Methods

### Cell culture

Nine triple negative breast cancer (TNBC) cell lines were used. HCC1806 (cat No. ATCC CRL-2335), HCC70 (cat No. ATCC CRL-2315), HCC1937 (cat No. ATCC CRL-2336), BT-549 (cat No. ATCC HTB-122), BT-20 (cat No. ATCC HTB-19), Hs578T (cat No. ATCC HTB-126), MDA-MB-231 (cat No. ATCC HTB-26), MDA-MB-157 (cat No. ATCC HTB-24), and MDA-MB-468 (cat No. ATCC HTB-132) were purchased from ATCC in 2016. BT-549 cell line was purchased from National Cancer Institute-Frederick cancer DCTD tumor/cell line repository. All cell lines were mycoplasma-free and used within the first 10 passages. HCC1806, HCC70, HCC1937, and BT-549 were cultured in RPMI-1640 medium. BT-20 was cultured in Eagle’s Minimum Essential Medium (EMEM). Hs578T, MDA-MB-231, and MDA-MB-157 were cultured in Dulbecco’s Modified Eagle Medium (DMEM). MDA-MB-468 was cultured in Leibovitz’s L-15 medium. Each medium was supplemented with 10% fetal bovine serum (FBS), 1% glutamine, and 1% antibiotic. One passage prior to migration experiments with chemical compounds, phenol red-free growth medium was used to culture the cells. Cells were cultured in T75 flasks in a humidified incubator at 37 °C with 5% CO_2_ until they formed a monolayer of about 90% confluent. Then, cells were dislodged using 3 ml of a 0.25% trypsin (Life Technologies) for 5–8 min in an incubator, collected with 6 ml of complete growth medium, and centrifuged down at 1000 rpm for 5 min. After removing the supernatant and re-suspending the cell pellet in 1 ml of complete medium, cells were counted with a hemocytometer.

### Test compounds

Fisetin, dactolisib, GSK1059615, BML-277, and WNK463 were purchased from Selleckchem. Quercetin was purchased from Sigma-Aldrich. Stock solutions of the compounds were prepared on the day of an experiment by dissolving the powder in dimethyl sulfoxide (DMSO). Stock solutions were kept at − 80 °C for long-term storage. Working concentrations were diluted from respective stock solutions using complete growth medium. Dilutions of fisetin and quercetin were prepared in phenol red-free media. All solutions were protected from light.

### Cell viability experiments

An AlamarBlue assay (Life Technologies) was used to determine viability of TNBC cells treated with the test compounds. The Alamar Blue reagent contains an active ingredient, resazurin, which is reduced by metabolically active cells to a fluorescent form, resorufin. The level of fluorescent signal correlates with cell viability. TNBC cells were seeded in a 96-well plate and maintained in a concentration range of 0–200 μM fisetin or quercetin for 48 h. Then, 10% of the total well volume of the AlamarBlue reagent was added to treated and non-treated (vehicle control) cells. After 6 h of incubating the cells at 37 °C, cell viability was determined by measuring the fluorescent signal using a plate reader (Synergy H1M, Biotek Instruments). Only those concentrations from each compound that resulted in a viability of greater than 90% were used in subsequent migration experiments.

### Preparation of aqueous two-phase system

Our cell migration assay used a polymeric aqueous two-phase system (ATPS) [17]. An ATPS was formed with 10.0% (w/v) polyethylene glycol (PEG), Mw 35 kDa, (Sigma-Aldrich) and 6.4% (w/v) dextran (DEX), Mw 500 kDa, (Pharmacosmos), as phase-forming polymers [18]. Both polymeric solutions were prepared in a phenol red-free complete growth medium 1 day prior to experiments with the phytochemicals. To facilitate dissolving the polymers, the solutions were vortexed and incubated in a water bath at 37 °C for 1 hour. The solutions were stored at 4 °C. While the DEX phase solution was used at this concentration, the aqueous PEG phase was diluted to 5.0% (w/v) after mixing with cell suspension, as explained below.

### Micropatterning of migration niche

We used ATPS to generate a cell-excluded migration niche [19]. Aqueous DEX phase drops of 1 μl volume were simultaneously printed in 96-well plates (Greiner Bio-One) using a robotic liquid handler (SRT Bravo, Agilent), one drop in the center of each well. The plate, with its lid covered, was kept inside a sterile biological safety cabinet for 24 h to allow the drops to dry. This protocol consistently gives dehydrated DEX phase drops of 2.00 ± 0.1 mm [18]. The density of each TNBC cell line was optimized to give a confluent layer of cells around the dehydrated drop. Cell suspensions were adjusted to a density of 3.5 × 10^5^ cells/ml of Hs578T, MDA-MB-231, BT-549, HCC1937, and BT-20, 4.5 × 10^5^ cells/ml of HCC1806 and HCC70, and 5.0 × 10^5^ cells/ml of MDA-MB-157. The suspensions were thoroughly mixed with an equal volume of the 10.0% (w/v) aqueous PEG phase. Each well was loaded with 100 μl of the resulting suspension of each cell line. This addition rehydrated the DEX phase drop. The interfacial tension between the two immiscible aqueous phases prevented the cells from entering into the drop such that cells could only adhere to the well surface around the drop [20]. This resulted in a well-defined cell-excluded gap within a monolayer in each well. After 12 h of incubation, the ATPS was replaced with growth medium only (vehicle control) or medium containing a test compound. The area of the cell-excluded gap within well plates averaged to 3.33 ± 0.09 mm^2^. A conventional scratch migration assay was performed for MDA-MB-468 cells in 6-well plates due to the difficulty with creating a micropatterned migration niche with these cells.

### Cell migration experiments

Migration experiments with the TNBC cells treated with fisetin and quercetin were performed for 48 h. A vehicle control condition was used with culture medium only and without any test compounds. Each condition had 8 replicates. Then, cells were stained with 2 μM Calcein AM (Life Technologies) for 30 min. Each well containing fluorescing cells was imaged using an inverted fluorescent microscope (AxioObserver, Zeiss) equipped with an AxioCam MRm camera (Zeiss). The initial gap area (*A*_*1*_) and final gap area after incubation (*A*_*2*_) were computed using ImageJ (NIH). The migration of cells over time was quantified as $\left(1-\frac{A_2}{A_1}\right)\ast 100$ [19]. The inhibition in migration of cancer cells by a chemical compound was quantified as the difference in migration of vehicle control cells and the migration of treated cells, i.e., migration inhibition= $\left(\frac{A_2(treatment)}{A_1}\right)-\left(\frac{A_{2(control)}}{A_1}\right)$. To study inhibitory effects of fisetin and quercetin against migration of TNBC cells, the largest concentration of each compound that resulted in a cell viability of at least 90% in cytotoxicity tests was used. In separate experiments, dose-dependent migration inhibition experiments were performed using GSK1059615 and WNK463 at concentrations of 62.5 nM, 125 nM, 250 nM, 500 nM, 1 μM, and 5 μM against 4 TNBC cell lines MDA-MB-231, MDA-MB-157, HCC1806, and BT-59. In addition, BML-277 was used to stimulate migration of these cells for 6 h, 12 h, and 24 h.

### 3D invasion assay

MDA-MB-157 and BT-549 cells were stained with 2 μM Calcein AM before harvesting the cells for spheroid formation. An ATPS technology was used to form spheroids [21]. Spheroids were suspended in an ice-cold 4 mg/ml solution of type I rat tail collagen (Corning) and then incubated at 37 °C for 30 min to allow collagen gelation. Invasion of the spheroids in collagen matrix was captured using fluorescent confocal microscopy (Nikon A1 confocal system) at a 10X magnification. FITC filter was used to capture images with a z-spacing of 20 μm. NIS Elements software was used for image acquisition. Z-projected images were reconstructed by collapsing the stacks using ImageJ (NIH) and total area of pixels was found using color thresholding of the resulting images.

### Zebrafish in vivo tumor metastasis and drug treatment assays

All procedures with zebrafish were conducted in accordance with NIH guidelines for the care and use of laboratory animals and approved by the Georgetown University Institutional Animal Care and Use Committee. The maximum tolerable dose (MTD) of fisetin for zebrafish was first determined by fisetin treatment (6.25 μM - 100 μM) in fish water for 7 days. A 0.5% DMSO treatment was used as vehicle control. Two days post fertilization (dpf), fish were arrayed in 24 well plates, 5 fish in each well, and scored for death and edema daily. A MTD of 100 μM for fisetin was determined where there was no death of fish and negligible edema due to drug exposure. For the evaluation of metastasis, cells were first labeled with a lipophilic CM-diI dye (Thermo Fisher) according to the manufacturer’s instructions. MDA-MB-157 and BT-549 cells were pre-treated with fisetin or 1% DMSO for 3 h during the labeling step, washed vigorously and injected in to the yolk sac of 2 dpf zebrafish embryos. Zebrafish embryos were injected with 100–200 labeled cancer cells into the yolk sac. Invasion of the vasculature was monitored as a surrogate of metastatic potential at 10x magnification using an Olympus IX-71 inverted microscope. Injected embryos were evaluated at 2–3 h post injection to discard embryos from analysis if they showed any cells in the vasculature as that would indicate improper injection. Properly injected embryos were arrayed in 96-well plates. Fish injected with cells were treated with 100 μM fisetin, whereas 0.5% DMSO treated fish were used as a vehicle control. Embryos were evaluated daily for tumor cell migration and health. With each of the cell lines, at least 200 fish were injected for the fisetin treatment group and 100 fish for the DMSO control group. Box plots of scores of fish were made using Statview 5.01 (SAS Institute, Cary, NC, USA). Paired student's t-test was performed on the data and *p* < 0.05 represented statistically significant differences. Transgenic zebrafish, *Tg (kdrl:grcfp)zn1*, expressing green reef coral fluorescent protein in the vascular endothelium, were used for tracking of cancer cells. This line, which was obtained from the Brant Weinstein Lab in National Institute of Child Health and Human Development, has been propagated at Georgetown University in our zebrafish facility for the past 15 years. Zebrafish embryos were euthanized by hypothermia following the conclusion of the experiments.

### Phospho-kinase array experiments

TNBC cells were treated with fisetin and quercetin at a 200 μM concentration for 6 h. A human phospho-kinase dot blot array (ARY003B; R&D Systems) was used to simultaneously detect the relative levels of phosphorylation of 43 human kinases and total amounts of 2 related proteins. An equal amount of cell lysate (300 μg) from each TNBC cell line was used for these experiments. The array was visualized using a FluorChem E Imaging System (ProteinSimple). All arrays of one experiment were exposed simultaneously. An adequate exposure time was chosen to capture the differences in protein kinase activity. A pixel density module in ImageJ was used to quantify phosphorylation levels of the proteins. The pixel density of the background signal was subtracted from the average of measured signal of a pair of dots for each protein on the array. The phosphorylated level of each protein in a treated group was determined by normalizing it to the pixel density value in the respective vehicle control group.

### Western blot experiments

Cells were washed with PBS and lysed in 500 μL of complete RIPA buffer (50 mM Tris-HCl, 150 mM NaCl, 1% NP-40, 0.5% sodium deoxycholate, and 0.1% SDS, pH 7.4 ± 0.2) with protease inhibitor (complete mini, Roche Diagnostics) and phosphatase inhibitor (Life Technologies). Total protein concentration was determined using a BCA quantification assay kit (Life Technologies). Equal amounts of protein were loaded onto a 4–15% gel (Biorad) for electrophoresis and the gel was transferred onto a nitrocellulose membrane by electroblotting. Membranes were blocked with 5% BSA (Sigma) for 1 h. Primary antibodies used were phospho-WNK1 (p-WNK1), WNK1, phospho-Akt (Ser473), and Akt (pan) (C67E7), all purchased form Cell Signaling Technology. Solutions of primary antibodies were prepared at concentrations recommended by the manufacturer. Membranes were incubated with the primary antibody solutions at 4 °C overnight. After repeated washing, membranes were incubated with a horseradish peroxidase (HRP)-conjugated secondary antibody for 1 h, followed by another round of repeated washing. Detection was carried out with an ECL chemiluminescence detection kit (GE Healthcare) using a FluorChem E imaging system.

### Statistical analysis

Statistical analysis was performed in Excel using a two-tailed distribution and heteroscedastic student’s t-test. For cell viability studies, *p*-values were obtained using viability of cells treated with a phytochemical and the vehicle control group. Statistical significance was defined as *p* < 0.05. An agglomerative hierarchical cluster analysis with the complete linkage method was used to assign the relative phosphorylation levels of phospho-kinases in TNBC cells into different clusters. The same analysis was used to cluster phospho-kinases of TNBC cells after treatment with fisetin or quercetin.

## Results

### Migration of TNBC cells

**Fig. 1:**
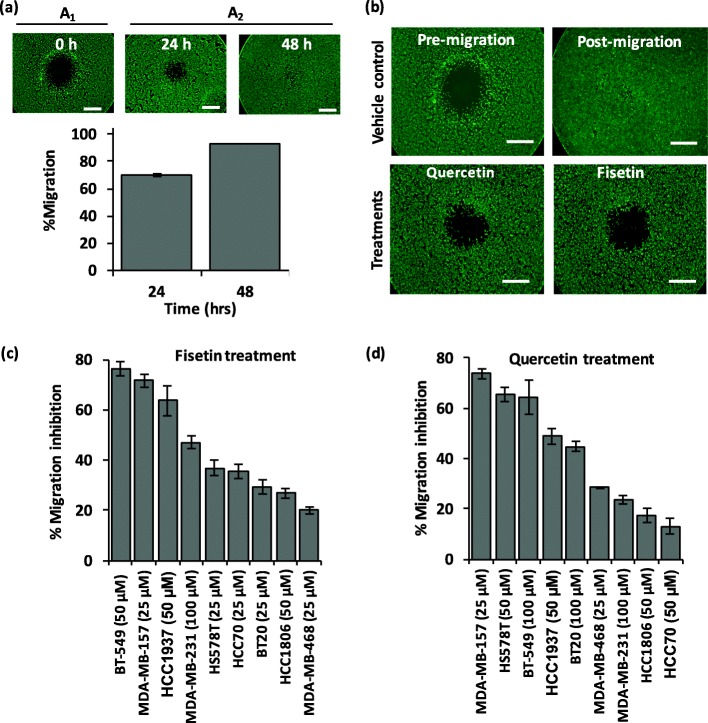
Phytochemicals inhibit migration of TNBC cells. **a** A micropatterned circular gap within a layer of BT-549 TNBC cells before migration (left, A_1_), after 24 h (middle, A_2_), and after 48 h (right, A_2_) of migration. Bar graph in (**a**) shows percent migration, i.e., 1-(A_2_/A_1_), of BT-549 cells for two different time points. Each bar represents the mean of 8 replicates and each error bar represents standard error from the mean. Scale bar in (**a**) is 1 mm. **b** Representative images of migration inhibition of BT-549 cells with fisetin and quercetin treatments compared to non-treated cells. Scale bar in (**b**) is 1 mm. Inhibition of migration of nine TNBC cell lines by (**c**) fisetin and (**d**) quercetin. Each bar represents the mean of 8 replicates and error bars are standard error from the mean. Concentrations shown on the x-axis are threshold concentrations of the compounds to maintain a viability of over 90% for each cell line

Selective partitioning of cells to the aqueous PEG phase resulted in adhesion of cells to the well bottom around the DEX phase drop and formation of circular gap (Fig. 1a, left). Over time, cells migrated into the gap and reduced the available area (Fig. 1a, middle and right). We quantified migration of TNBC cells using the gap closure. Figure 1a shows a typical time-dependent gap closure for BT-549 cells that occupied 69 and 93% of the gap after 24 h and 48 h, respectively. We have previously demonstrated that while cell proliferation within the monolayer of cells play a supportive role for the forward movement of the cells at the leading edge, the gap closure primarily results from cell migration [18]. The nine TNBC cell lines migrated at different rates (Additional file 1: Figure S1). Mesenchymal-like (ML) TNBC cells showed an overall greater migration than basal-like (BL) TNBC cells, with an exception of HCC1937 that is a basal subtype TNBC but migrated faster than all 4 ML cells and occupied ~ 93% of the gap only after 24 h (Additional file 1: Figure S2). BT-20 cells, an unclassified TNBC subtype, were the slowest and only occupied 35% of the gap after 48 h. Based on this result, we selected a 48 h-window to test migration inhibition potential of the chemical compounds.

### Inhibitory effects of phytochemicals on migration and matrix invasion of TNBC cells

In a previous study, we examined the potential of 20 phytochemicals to block migration of two metastatic TNBC cell lines and identified fisetin and quercetin as the most effective compounds [13]. To establish whether these phytochemicals are broadly active against migration of TNBC cells, we tested them against nine TNBC cell lines. Prior to these experiments, we performed dose-dependent cell viability tests to determine threshold concentrations that give over 90% viability for each TNBC cell line during a 48-h incubation to match the duration of subsequent migration experiments (Additional file 1: Figure S3a-b). We also visually examined morphology of the cells under treatments. Interestingly, cells in the vehicle control group had a spindle ML shape, whereas cells treated with fisetin or quercetin were less elongated and had a cuboidal epithelial-like shape (Additional file 1: Figure S3c-e). This shift in morphology was more prominent in the ML TNBC cells than in the BL TNBC cells.

We used the threshold concentrations to investigate anti-migratory effects of fisetin and quercetin on TNBC cells by calculating inhibition of migration using pre-migration and post-migration images of cells from vehicle control and treated groups, as shown for BT-549 cells (Fig. 1b). Fisetin and quercetin were effective and reduced migration of the nine TNBC cells in a range of 20 to 76% (Fig. 1c) and 13 to 74% (Fig. 1d), respectively. The results also suggested a subtype-dependent inhibition of migration by the compounds. Fisetin treatment inhibited migration of all ML TNBC cells more effectively than the BL cells, except for the BL HCC1937 cells that ranked third in terms of migration inhibition (Fig. 1c). Similarly, the effect of quercetin treatment to inhibit migration of ML TNBC cells was more pronounced than the BL cells, except for the BL HCC1937 cells that ranked fourth and the ML MDA-MB-231 cells that ranked seventh among all TNBC cells (Fig. 1d).

**Fig. 2:**
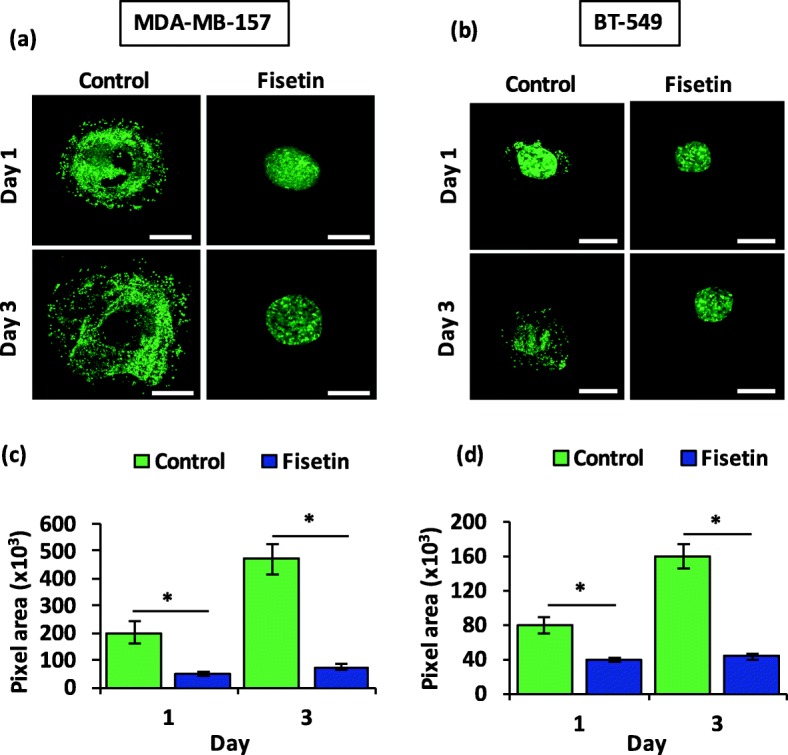
Fisetin inhibits collagen invasion of TNBC cells. Z-projected images of spheroids of (**a**) MDA-MB-157 and (**b**) BT-549 cells in collagen hydrogel without and with 100 μM fisetin treatment for 24 and 72 h. Scale bar is 300 μm. Total pixels area occupied by (**c**) MDA- MB-157 and (**d**) BT-549 cells in the collagen matrix. *p < 0.001

Next, we asked whether phytochemicals have inhibitory effects against TNBC cell migration and invasion in 3D environments. We formed spheroids of highly motile ML MDA-MB-157 and BT-549 cancer cells, embedded them in a type I collagen matrix of ~ 2.7 kPa elastic modulus to mimic human breast tumors [22], treated them with fisetin, and performed confocal imaging of cells. Fisetin suppressed collagen invasion of MDA-MB-157 cells by four and six folds after 24 and 72 h of treatment, respectively, compared to the vehicle control group (Fig. 2a-b). Similarly, fisetin treatment reduced the matrix invasion of BT-549 spheroids by two and four folds after 24 and 72 h, respectively (Fig. 2c-d).

### Phytochemical treatment inhibition of metastasis in zebrafish

**Fig. 3:**
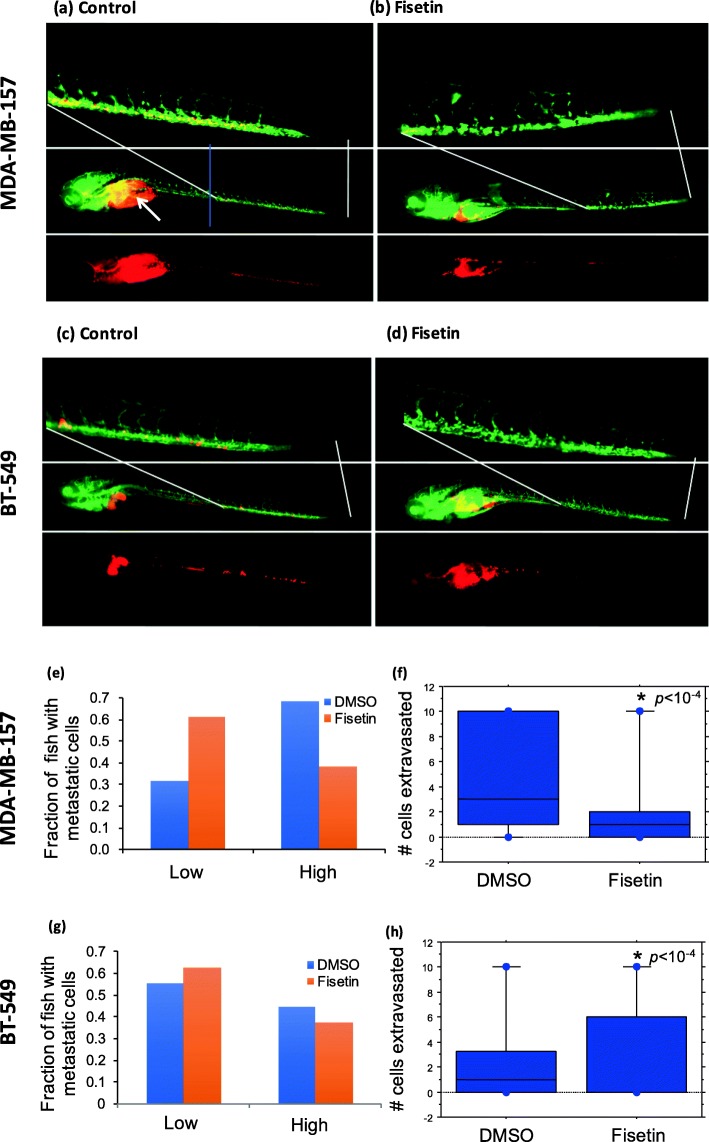
Fisetin treatment reduces TNBC metastasis in vivo. MDA-MB-157 and BT-549 cells were injected into two-day post-fertilization zebrafish embryos. Fish were treated either with 0.5% DMSO or with 100 μM Fisetin. Cells were allowed to migrate to the tail for 4 days. **a**, **c** Representative images of DMSO-treated fish with intravasated cells in to the tail. Green shows fish vasculature and red represents cancer cells. **b**, **d** Representative images of fisetin-treated fish. **e**, **g** Fish with less than two cells in the tail were grouped as low and fish equal to or more than two cells in the tail were grouped as high. **f**, **h** Box plots of extravasated cells in vehicle control compared to the treated groups. Note that in fisetin-treated fish with BT-549 cells, the median is at zero. A blue line in the middle panel of (**a**) indicates the cut-point for separating the tail from the rest of the fish. A white arrow in the same panel indicates the site of injection for tumor cells in the yolk sac. Top panels are high-resolution images for tails to highlight the migrated tumor cells in the tail region. Bottom panel shows the location of tumor cells (red) in the fish

We aimed to determine whether phytochemicals inhibit cell migration and metastasis in vivo. We used a zebrafish tumor metastasis model because it has been validated for studies of human cancer cell metastasis and allows high throughput testing of a large number of fish with convenient imaging and analysis [23]. Consistent with our in vitro experiments above, we treated fish with fisetin to evaluate inhibition of metastasis of MDA-MB-157 and BT-549 cells. Injection of MDA-MB-157 cells into the yolk sac of 2 dpf stage embryos resulted in rapid movement of a small fraction of cells to the tail and head regions as early as 4 days post injection in the DMSO-treated group (Fig. 3a). Cells intravasated into the dorsal aorta, and more than 5 cells were observed throughout the tail. We obtained similar results with BT-549 cells injected into 2 dpf zebrafish embryos under DMSO treatment with more than 5 cells intravasating into the main blood vessel in the tail 4 days post injection (Fig. 3c). These results established the metastatic potential of the TNBC cell lines in zebrafish. As a measure of metastasis, we scored the number of embryos with cells that migrated to the tail after 4 days and quantified the number of fish with metastasis if there was more than one cancer cell in the vasculature [24]. With MDA-MB-157 and BT-549 cells, 69 and 45% of fish in vehicle control groups developed metastasis (data bars labeled High in Fig. 3e, g). When we treated fish with fisetin, metastasis of cells to the tail significantly reduced (*p* < 10^− 4^) (Fig. 3b, d and Fig. 3f, h). Fisetin more effectively inhibited migration of MDA-MB-157 cells than BT-549 cells, i.e., by 1.77-fold and 1.20-fold, relative to their respective vehicle controls. Overall, this study established the potential of these phytochemicals against TNBC cell metastasis.

### Molecular analysis of TNBC cells under phytochemicals treatment

**Fig. 4:**
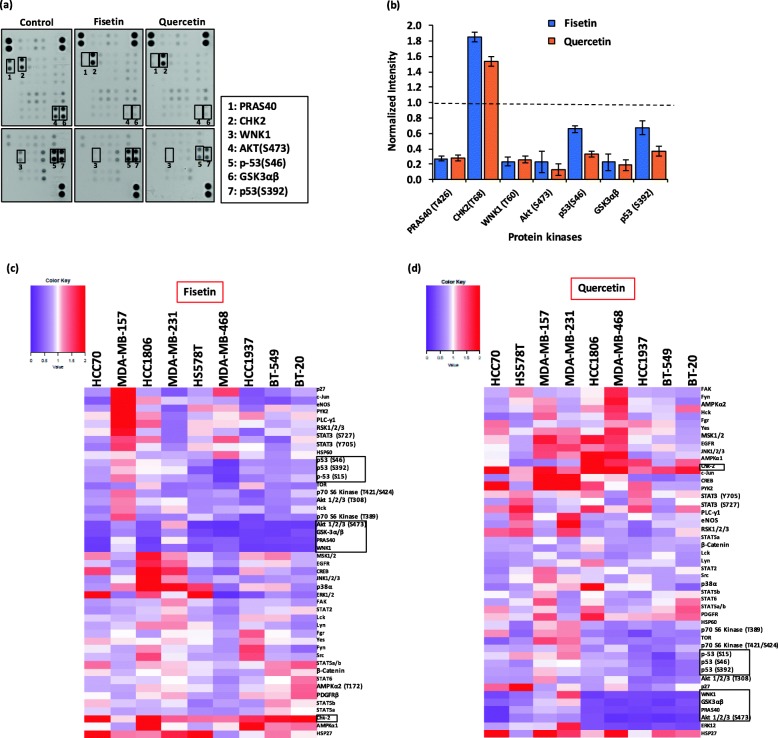
Molecular effects of phytochemicals on TNBC cells. **a** Representative human phospho-kinase blot array of BT-549 cells under no treatment (left), fisetin treatment (middle), and quercetin treatment (right). The kinases boxed and numbered in the blots represent major signaling protein targets of the phytochemicals. **b** Quantified normalized dot blot intensity of multiple kinases from fisetin and quercetin treatments of BT-549 cells. Error bars represent standard error from the mean (*n* = 2). Heatmaps of normalized phosphorylated levels of 43 protein kinases and 2 related proteins in nine TNBC cell lines treated with (**c**) 200 μM fisetin and (**d**) 200 μM quercetin. Phosphorylated level of each protein from a treated group was normalized by that from the corresponding vehicle control group to generate the heatmaps. Signaling molecules highlighted in boxes showed significant changes in phosphorylation by fisetin and quercetin treatments

Oncogenic kinase signaling pathways such as PI3K/AKT/mTOR and MAPK have established roles in migration and metastasis of TNBC cells [25, 26]. Thus, we first determined baseline protein kinase activities in untreated TNBC cells using a human phospho-kinase array. This analysis resulted in four major clusters (Additional file 1: Figure S4). The two clusters in the top and bottom of this heatmap show highly active kinases in the TNBC cells. The bottom cluster consists of proteins of PI3K/AKT/mTOR pathway PRAS40 and AKT(S473), the multifunctional kinase GSK3α/β, and phospho-p53(S46 and S392). The top cluster contains active ERK1/2, WNK-1, HSP60, p53(S15), and CHK-2. Due to the role of these signaling molecules in TNBC cell migration, we aimed to identify if anti-migratory effects of fisetin and quercetin were due to disrupting the activity of these kinases. We conducted the phospho-kinase array experiment with all nine TNBC cells after treatments with a non-toxic concentration of fisetin and quercetin (Additional file 1: Figure S5). Figure 4a displays a representative blot array for vehicle control and treated BT-549 cells. We quantified and normalized the phosphorylation status of various proteins in a treated group to that of the respective vehicle control group. Figure 4b shows the normalized phosphorylated levels of proteins in these clusters in treated BT-549 cells. We subjected the results with all nine TNBC cell lines to a cluster analysis. Figure 4c-d show the resulting heatmaps for fisetin and quercetin treatments.

The phytochemicals significantly targeted signaling of PI3K/AKT/mTOR pathway across the TNBC cell lines. Fisetin reduced p-AKT(S473) and p-AKT(T308) levels by over 70% and 25–54%, respectively. Additionally, fisetin downregulated activities of downstream kinases of this pathway, including m-TOR(S2448) by 40–61%, P70S6K(T398) by 10–84%, and P70S6K(T421/S424) by 23–63%. The reduced activities of these proteins occurred in at least seven of the TNBC cell lines. Quercetin significantly reduced p-AKT(S473) levels in all TNBC cells by 27–88% and inhibited p-AKT(T308) by 25–55% in seven of the cell lines, leading to reduced activities of P70S6K and m-TOR in these cells. Significant inhibition of AKT also decreased active PRAS40 levels by 14–85% in all nine TNBC cells. Fisetin and quercetin also reduced phosphorylation of GSK3α/β, which is a substrate of PI3K/AKT/mTOR pathway, in the TNBC cell lines by 40–86% and 12–82%, respectively.

**Fig. 5:**
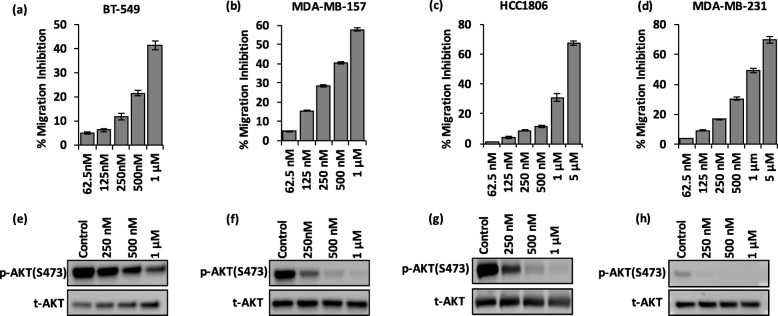
GSK1059615 dose-dependently inhibits migration of TNBC cells. **a**-**d** Migration inhibition of TNBC cells after 48 h treatment with GSK1059615. Maximum concentration used for each cell line was limited by toxicity. **e**-**h** Western blots of p-AKT and t-AKT for TNBC cells under GSK1059615 treatment. Note that the y-axis scale is different in panels (**a**-**d**)

To establish that fisetin and quercetin suppression of PI3K/AKT pathway signaling contributes to inhibition of migration of TNBC cells, we treated cells with non-toxic concentrations of an inhibitor of this pathway, GSK1059615. We selected four cell lines for this study, BT-549, MDA-MB-157, HCC1806, and MDA-MB-231, due to their differential sensitivities to inhibition of migration by fisetin and quercetin (Fig. 1c-d). This treatment dose-dependently reduced migration of all four TNBC cells (Fig. 5a-d). GSK1059615 was less effective than the phytochemicals against BT-549 and MDA-MB-157 cells, suggesting that other mechanisms additionally regulate migration of these cells. With HCC1806 and MDA-MB-231 cells, GSK1059615 generated a significantly greater anti-migratory effect than the phytochemicals, indicating strong dependency of these cells on PI3K/AKT pathway for cell migration. Our molecular analysis showed a dose-dependent downregulation of p-AKT (S473) in the cells (Fig. 5e-h), consistent with the cell migration results. Overall, inhibition of PI3K/AKT pathway by fisetin and quercetin reduced migration of TNBC cells but the extent depended on the significance of this pathway in migration of specific cell lines.

We also observed a significant reduction in p-WNK1 levels (> 50%) by fisetin and quercetin treatments in the TNBC cells. WNK1 has been suggested as a substrate of AKT [27], with a potential role in regulating EMT and migration of glioblastoma and non-small cell lung cancer cells [28, 29]. We showed that WNK1 activity depends on AKT by treating four TNBC cell lines with GSK1059615, which dose-dependently reduced phosphorylated WNK1 levels in all four cell lines (Additional file 1: Figure S6). Our combination treatment using a pan-WNK inhibitor, WNK463 (0.5 μM), and GSK1059615 (1 μM) did not result in a significant decrease of the p-AKT or p-WNK1 compared to the single-agent treatment with GSK1059615. Both treatments blocked p-AKT by ~ 98% and p-WNK1 by ~ 79% (Additional file 1: Figure S7). These results suggest that WNK1 activity is regulated by AKT. However, the use of an inhibitor specific to WNK1 or its direct targeting through gene silencing is necessary to establish whether WNK1 has an independent role from AKT on migration of TNBC cells.

**Fig. 6:**
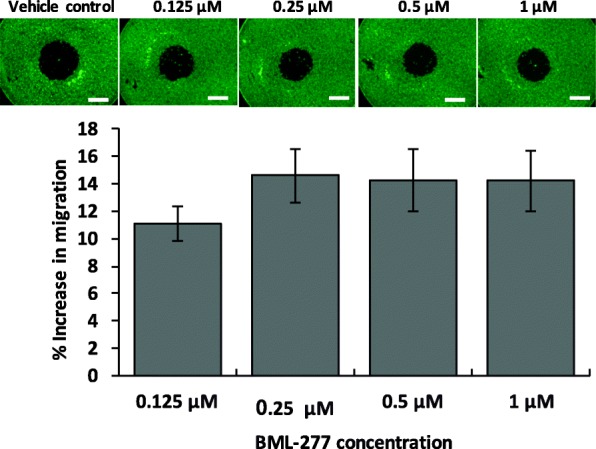
Inhibition of checkpoint kinase 2 (CHK2) promotes migration of TNBC cells. Dose-depending inhibition of CHK2 using BML-277 increases migration of MDA-MB-231 cells. Each bar represents a mean of 8 samples and error bars represent standard error from the mean. Scale bar is 1 mm

Activation of CHK2 has been associated with a decrease in invasive potential of cells in p53 mutant cancers [30]. Interestingly, fisetin and quercetin significantly increased pCHK2 levels in all nine TNBC cells by 6–170% and 13–187%, respectively. These results led us to the hypothesis that if effects of fisetin and quercetin against migration of TNBC cells were in part due to increased CHK2 activity, inhibiting it would promote cell migration. First, we tested this hypothesis using a CHK2 inhibitor (BML-277) against MDA-MB-231 cells. We temporally monitored migration of cells treated with 0.125 μM - 1 μM of the inhibitor. At 6 h, the vehicle control cells migrated only by 2.54%, whereas BML-277 treatment of the cells led to a dose-dependent increase in cell migration by up to 5.8-fold (Fig. 6). Next, we validated that the role of CHK2 was not specific to MDA-MB-231 cells by performing migration experiments with BT-549, MDA-MB-157, and HCC1806 cells. Treatment with BML-277 increased cell migration by 4.0, 4.4, and 2.0 folds in these three cell lines, respectively (Additional file 1: Figure S8). Therefore, increased activity of CHK2 by phytochemicals is an additional mechanism for inhibiting migration of TNBC cells. In addition, the phytochemicals targeted mutant p53 in most TNBC cells. Fisetin reduced phosphorylated p53 levels by 19–75% (S392), 17–81% (S46), and 12–79% (S15), whereas quercetin inhibited p53 phosphorylation by 18–63% (S392), 7–67% (S46), and 17–76% (S15). These phosphorylation sites on p53 encompass several different upstream kinases, consistent with fisetin and quercetin inhibiting multiple pathways.

Other than the above signaling pathways and molecules that were most commonly targeted by fisetin and quercetin treatments, we observed cell-specific changes in the activities of several other pathways frequently implicated in cell migration and invasion. 1) MAPK pathway is a major driver of progression of various cancers including TNBC [31]. Fisetin and quercetin reduced activities of signaling molecules in this pathway (ERK1/2, JNK1/2/3, p38α) in several TNBC cell lines, consistent with our previous preliminary study that showed fisetin downregulated p-ERK levels in MDA-MB-157 and MDA-MB-231 by 49 and 5% relative to the respective vehicle controls [13]. 2) Different STAT proteins including STAT3 and STAT5 have been implicated in cancer invasion and metastasis [32, 33]. Fisetin reduced active levels of STAT3 by 30–60% in at least three TNBC cells and STAT5A by 20–30% in seven TNBC cell lines, and quercetin moderately suppressed various STAT kinases in four cell lines. (3) The nonreceptor protein tyrosine kinase SRC plays a crucial role in signal transduction pathways involved in cell motility [34]. Among SRC kinases, fisetin and quercetin respectively reduced active Hck levels by 10–50% and 25–40% in seven of the TNBC cells and also moderately downregulated all seven SRC kinases in a few of the cell lines. These cell-specific activities of the phytochemicals potentially reflect differences in the extent and significance of mutations that regulate motility of the specific TNBC cells.

## Discussion

TNBC has the highest prevalence of metastasis and the poorest prognosis and survival among different subtypes of breast cancer. With the lack of targeted and endocrine therapies for TNBC, strategies to tackle metastasis can potentially enhance survival of patients. However, existing cytotoxic and cytostatic agents used to treat TNBC are primarily designed to kill cancer cells and shrink tumors without directly targeting key events in metastasis. There is currently a pressing need for novel chemicals that target processes central to metastatic disease including migration and invasion of cancer cells. We demonstrated the feasibility of this approach through a comprehensive set of phenotypic and molecular studies using fisetin and quercetin. These phytochemicals effectively reduced migration of a panel of TNBC cells, with a greater effect against mesenchymal-like (ML) subtype than basal-like subtype cells. Only two of the cell lines (ML MDA-MB-231 and BL HCC1937) did not follow this trend. Importantly, the treatments altered the morphology of metastatic TNBC cells from a spindle shape to a cuboidal shape, suggesting transition from a mesenchymal to an epithelial phenotype (mesenchymal-to-epithelial transition, MET). This is consistent with the reduced migration and ECM invasion of TNBC cells and inhibition of metastasis in zebrafish, although detailed molecular studies are required to substantiate whether these treatments induce MET. Overall, these studies established the potential of fisetin and quercetin as therapeutic agents against TNBC metastasis.

At a molecular level, fisetin and quercetin broadly targeted various signaling molecules. Their most common targets were components of the PI3K/AKT pathway, including AKT1/2/3, PRAS40, and mTOR, and additionally, GSK3, WNK-1, p53, and CHK-2. This is consistent with studies from cell-based assays and mouse models that showed a major role for PI3K/AKT/mTOR pathway in promoting migration and invasion of TNBC cells [35]. The treatments also reduced GSK3α/β phosphorylation in the TNBC cells, resulting in increased GSK activity [36]. While GSK3 activity is critical for normal cells to maintain an epithelial phenotype [37], its deregulation in cancer cells promotes activities of transcription factors such as AP-1 frequently implicated in cancer [38], and stability of Snail protein to facilitate EMT and invasion of cancer cells [36, 39]. Conversely, active GSK3 renders various EMT transcription factors such as Slug and Snail inactive and inhibits cell migration. Pharmacological inhibition of GSK3 generates various anti-tumor effects [40]. Significant suppression of GSK3α/β phosphorylation in TNBC cells by fisetin and quercetin is consistent with the role of this protein kinase in cancer cell motility and metastasis.

In agreement with our results of inhibitory role of CHK-2 in cell migration, suppression of YAP1 (an EMT marker) upregulated p-CHK2 in TNBC cells with mutant p53 [30], and upregulation of CHK2 activity by a natural podophyllotoxin analog, 4′-demethyl-deoxypodophyllotoxin glucoside (4DPG), abrogated invasion of TNBC cells by restraining Twist1 [41]. Additionally, our finding that fisetin and quercetin treatments reduce phosphorylation of mutant p53 to interfere with TNBC cell migration is consistent with the role of gain-of-function mutant p53 in tumorigenesis, migration, invasion, and metastasis of cancer cells, through various mechanisms such as binding to and inactivating p63 and the Mre11 nuclease [42–44]. The inhibitory effect of reduced mutant p53 activity on TNBC cell migration in our study is also consistent with reduced migration and invasion of TNBC cells both in vitro and in mice through RNAi-mediated knockdown of mutant p53 [45]. Considering that all nine TNBC cells used in our study have p53 mutations, our results suggest that reduced activity of mutant p53 in part accounts for the inhibition of TNBC cell migration by fisetin and quercetin.

Apart from these proteins, we observed cell-specific effects of the phytochemicals on various signaling molecules that can be explained by key mutations in these cells (Additional file 1: Figure S1) [46], such as in the following examples:

1. BT-549 cells have a PTEN mutation. Genomics and proteomics analyses and histological examination of breast tumors showed an association between PTEN mutation, EMT, and metastasis driven by activated PI3K/AKT and MAPK signaling [47, 48]. This is consistent with our findings that fisetin and quercetin significantly downregulated activities of signaling molecules of these pathways in BT-549 cells, including AKT (S473/T308), TOR, P70S6K(T421/S424 and T389), and PRAS40 by 37–77% and 40–87%, respectively, GSK3α/β activity by 77 and 81%, and p-ERK by 30 and 66%.
2. MDA-MB-157 cells contain a mutation in Neurofibromatosis type 1 (NF1). Truncations and deletions in NF1 activate RAS and its downstream pathways RAF/MEK/ERK and PI3K/AKT [49, 50], both of which are prominent drivers of breast cancer metastasis. Fisetin downregulated p-ERK by 50% and several kinases of PI3K/AKT pathway such as p-AKT and mTOR by 30 and 40%. Quercetin also downregulated p-ERK by 20% and kinases of PI3K/AKT pathway such as p-AKT(S473) by 19%, PS0S6K (T389) by 14%, and PRAS40 by 14% respectively.
3. Among several other mutations, HCC1937 cells are BRCA1-mutant. Normally, BRCA1 negatively regulates the PI3K/AKT pathway through ubiquitination of p-AKT, leading to its degradation [51]. Activation of this pathway in BRCA1-deficient breast cancer leads to EMT, cell motility, and tumor progression and metastasis [52, 53]. We found that fisetin downregulated signaling molecules of PI3K/AKT pathway including AKT(S473/T308), P70S6K(T421/S424 and T389), and PRAS40 by 54–75%. Similarly, quercetin downregulated AKT(S473), AKT(T308), mTOR, P70S6K(T389), P70S6K(T421/S424), and PRAS40 by 40–82%. Additionally, fisetin and quercetin treatments downregulated GSK3α/β by 76 and 73%, mutant p53 by 19–42% and 33–49%, and p-ERK by 64 and 76%, respectively.

Overall, our results are consistent with multiple mechanisms that drive migration and metastasis of these TNBC cells and highlight the efficacy of fisetin and quercetin against various oncogenic signaling events. We emphasize that although the lack of specificity against a single target may be perceived undesirable, these compounds broadly impact multiple oncogenic proteins in different pathways that cannot be achieved with standard chemotherapeutics or without using multiple molecular inhibitors. Additionally, the use of a molecular inhibitor against a single target often results in feedback activation of other oncogenic pathways, rendering the treatment ineffective. For example, inhibition of the MAPK pathway almost always leads to activation of the PI3K pathway or overexpression of certain receptor tyrosine kinases such as EGFR and IGFR to activate other oncogenic kinase pathways. Treatment with a combination of several compounds is a strategy to address the problem, but this approach often leads to excessive toxicity. Therefore, simultaneous targeting of multiple pathways by fisetin or quercetin is a major benefit to prevent or reduce feedback signaling of pathways in TNBC cells. In addition to their role in cell migration, protein kinases regulate other processes such as cancer cell proliferation, survival, metabolism, and apoptosis [54]. While we comprehensively evaluated the impact of targeting protein kinases by fisetin and quercetin on TNBC cell migration and invasion, future studies should explore how these phytochemicals regulate other processes in TNBC and other cancers.

## Conclusions

Using our high throughput cell migration technology, we demonstrated that fisetin and quercetin effectively inhibited migration and matrix invasion of TNBC cells and significantly reduced metastasis in zebrafish. Our comprehensive analysis of an array of phospho-kinases showed high baseline activity levels of various oncogenic protein kinases in these cells. Treatments with the phytochemicals generated broad effects at a molecular level. The most common targets of fisetin and quercetin were components of PI3K/AKT pathway and its substrates, consistent with the significant inhibition of migration and invasion of several TNBC cell lines with constitutive activity of this pathway. Therefore, these compounds may significantly benefit cancers primarily driven by dysregulated PI3K/AKT activities. Additionally, fisetin and quercetin reduced activities of distinct signaling molecules cell-specifically, likely due to the role of the specific protein kinases in motility of the cells. Due to their broad effects on multiple signaling molecules in cancer cells, these phytochemicals may benefit targeting of several signaling events using only one compound, albeit without specificity of combinations of molecular inhibitors but without the associated toxicities. The use of fisetin and quercetin with tumor models composed of patient-derived cells will help elucidate their translational potential as therapeutic compounds.

## Additional file

**Additional file 1:** **Table S1.** Coordinates of protein kinases in the phospho-kinase dot blot array. **Figure S1.** (a) Migration of TNBC cells into the cell-excluded gap after 48 hrs without any treatment. (b) Subtypes of TNBC cell lines and their major mutations. **Figure S2.** Basal-like HCC1937 cells migrated into and occupied the gap almost within 24 hrs. Scale bar is 1 mm. **Figure S3.** Cytotoxicity analysis TNBC cells treated with (a) fisetin and (b) quercetin. Each data point represents the mean of 8 samples and error bars represent standard error from the mean. (c-e) Representative images of Hs578T show that the spindle-like mesenchymal morphology of cells changes to epithelial cell morphology with fisetin and quercetin treatments. Note that with fisetin treatment of HCC1806 cells and quercetin treatment of HCC1937 cells, a sigmoidal curve could not be fitted to the dose response data. **Figure S4.** Baseline relative phosphorylation of 43 protein kinases and 2 related signaling proteins in nine TNBC cell lines without any treatments. The hierarchical clustering identified 4 major clusters: Cluster 1 is highlighted with a red box (high baseline activity), Cluster 2 is highlighted with an orange box (high to moderate baseline activity), Cluster 3 is highlighted with a green box (moderate baseline activity), and Cluster 4 is highlighted with blue box (low baseline activity). **Figure S5.** Fisetin and quercetin treatments are non-cytotoxic to TNBC cells. Viability of nine TNBC cell lines after treatments with (a) 200 µM fisetin and (b) 200 µM quercetin for 6 hours. **Figure S6.** GSK1059615 treatment dose-dependently downregulated p-WNK1. (a-d) Western blots of p-WNK1 and t-WNK in TNBC cells treated with GSK1059615 for 6 hrs. **Figure S7.** Combination treatment of TNBC cells with GSK1059615 and WNK463 inhibitors produced an additive effect, suggesting that p-WNK1 is a p-AKT effector. (a) Western blot for single agent and combination treatments for 6 hrs. (b-c) Levels of p-AKT/t-AKT and p-WNK1/t-WNK1 in HCC1806 cells, respectively. ns represents lack of statistically significant difference. **Figure S8.** CHK2 inhibition promoted migration of different TNBC cells. Images of cell migration (a-c) without any treatment and (d-f) treatments with BML-277. Scale bar is 1 mm. (g) Quantified increased migration of TNBC cells by CHK2 inhibition. Each bar represents a mean of 8 samples, and error bars represent standard error from mean.

## Data Availability

All analyzed data are included in this published article and its supplementary information file. The original data are available upon request to the corresponding author.

## Abbreviations

BL: Basal-like
DEX: Dextran
ML: Mesenchymal-like
PEG: Polyethylene glycol
TNBC: Triple negative breast cancer

## References

[1] Stuelten CH, Parent CA, Montell DJ (2018). Cell motility in cancer invasion and metastasis: insights from simple model organisms. Nat Rev Cancer.

[2] Lambert AW, Pattabiraman DR, Weinberg RA (2017). Emerging biological principles of metastasis. Cell.

[3] Clark AG, Vignjevic DM (2015). Modes of cancer cell invasion and the role of the microenvironment. Curr Opin Cell Biol.

[4] Yamaguchi H, Condeelis J (2007). Regulation of the actin cytoskeleton in cancer cell migration and invasion. Biochim Biophys Acta.

[5] Denais CM, Gilbert RM, Isermann P, McGregor AL, te Lindert M, Weigelin B, Davidson PM, Friedl P, Wolf K, Lammerding J (2016). Nuclear envelope rupture and repair during cancer cell migration. Science.

[6] Friedl P, Alexander S (2011). Cancer invasion and the microenvironment: plasticity and reciprocity. Cell.

[7] Wolf K, Wu YI, Liu Y, Geiger J, Tam E, Overall C, Stack MS, Friedl P (2007). Multi-step pericellular proteolysis controls the transition from individual to collective cancer cell invasion. Nat Cell Biol.

[8] Gluz O, Liedtke C, Gottschalk N, Pusztai L, Nitz U, Harbeck N (2009). Triple-negative breast cancer-current status and future directions. Ann Oncol.

[9] Koboldt DC, Fulton RS, McLellan MD, Schmidt H, Kalicki-Veizer J, McMichael JF, Fulton LL, Dooling DJ, Ding L, Mardis ER (2012). Comprehensive molecular portraits of human breast tumours. Nature.

[10] Shen M, Jiang YZ, Wei Y, Ell B, Sheng X, Esposito M, Kang J, Hang X, Zheng H, Rowicki M (2019). Tinagl1 suppresses triple-negative breast cancer progression and metastasis by simultaneously inhibiting integrin/FAK and EGFR signaling. Cancer Cell.

[11] Chen L, Yang SY, Jakoncic J, Zhang JJ, Huang XY (2010). Migrastatin analogues target fascin to block tumour metastasis. Nature.

[12] Yoon YJ, Han YM, Choi J, Lee YJ, Yun J, Lee SK, Lee CW, Kang JS, Chi SW, Moon JH (2019). Benproperine, an ARPC2 inhibitor, suppresses cancer cell migration and tumor metastasis. Biochem Pharmacol.

[13] Ham SL, Nasrollahi S, Shah KN, Soltisz A, Paruchuri S, Yun YH, Luker GD, Bishayee A, Tavana H (2015). Phytochemicals potently inhibit migration of metastatic breast cancer cells. Integr Biol.

[14] Liou GY, Storz P (2010). Reactive oxygen species in cancer. Free Radic Res.

[15] Saini KS, Loi S, de Azambuja E, Metzger-Filho O, Saini ML, Ignatiadis M, Dancey JE, Piccart-Gebhart MJ (2013). Targeting the PI3K/AKT/mTOR and Raf/MEK/ERK pathways in the treatment of breast cancer. Cancer Treat Rev.

[16] Shimizu T, Tolcher AW, Papadopoulos KP, Beeram M, Rasco DW, Smith LS, Gunn S, Smetzer L, Mays TA, Kaiser B (2012). The clinical effect of the dual-targeting strategy involving PI3K/AKT/mTOR and RAS/MEK/ERK pathways in patients with advanced cancer. Clin Cancer Res.

[17] Tavana H, Jovic A, Mosadegh B, Lee QY, Liu X, Luker KE, Luker GD, Weiss SJ, Takayama S (2009). Nanolitre liquid patterning in aqueous environments for spatially defined reagent delivery to mammalian cells. Nat Mater.

[18] Lemmo S, Nasrollahi S, Tavana H (2014). Aqueous biphasic cancer cell migration assay enables robust, high-throughput screening of anti-cancer compounds. Biotechnol J.

[19] Tavana H, Kaylan K, Bersano-Begey T, Luker KE, Luker GD, Takayama S (2011). Rehydration of polymeric, aqueous, biphasic system facilitates high throughput cell exclusion patterning for cell migration studies. Adv Funct Mater.

[20] Atefi E, Joshi R, Mann JA, Tavana H (2015). Interfacial tension effect on cell partition in aqueous two-phase systems. ACS Appl Mater Interfaces.

[21] Atefi E, Lemmo S, Fyffe D, Luker GD, Tavana H (2014). High throughput, polymeric aqueous two-phase printing of tumor spheroids. Adv Funct Mater.

[22] Plodinec M, Loparic M, Monnier CA, Obermann EC, Zanetti-Dallenbach R, Oertle P, Hyotyla JT, Aebi U, Bentires-Alj M, Lim RY (2012). The nanomechanical signature of breast cancer. Nat Nanotechnol.

[23] Teng Y, Xie XY, Walker S, White DT, Mumm JS, Cowell JK (2013). Evaluating human cancer cell metastasis in zebrafish. BMC Cancer.

[24] Chen C, Choudhury S, Wangsa D, Lescott CJ, Wilkins DJ, Sripadhan P, Liu XF, Wangsa D, Ried T, Moskaluk C (2017). A multiplex preclinical model for adenoid cystic carcinoma of the salivary gland identifies regorafenib as a potential therapeutic drug. Sci Rep.

[25] Liu H, Murphy CJ, Karreth FA, Emdal KB, White FM, Elemento O (2018). Identifying and targeting sporadic oncogenic genetic aberrations in mouse models of triple-negative breast cancer. Cancer Discov.

[26] Costa RLB, Han HS, Gradishar WJ (2018). Targeting the PI3K/AKT/mTOR pathway in triple-negative breast cancer: a review. Breast Cancer Res Treat.

[27] Jiang ZY, Zhou QL, Holik J, Patel S, Leszyk J, Coleman K, Chouinard M, Czech MP (2005). Identification of WNK1 as a substrate of Akt/protein kinase B and a negative regulator of insulin-stimulated mitogenesis in 3T3-L1 cells. J Biol Chem.

[28] Hung JY, Yen MC, Jian SF, Wu CY, Chang WA, Liu KT, Hsu YL, Chong IW, Kuo PL (2017). Secreted protein acidic and rich in cysteine (SPARC) induces cell migration and epithelial mesenchymal transition through WNK1/snail in non-small cell lung cancer. Oncotarget.

[29] Zhu W, Begum G, Pointer K, Clark PA, Yang SS, Lin SH, Kahle KT, Kuo JS, Sun DD (2014). WNK1-OSR1 kinase-mediated phospho-activation of Na+−K+-2Cl(−) cotransporter facilitates glioma migration. Mol Cancer.

[30] Andrade D, Mehta M, Griffith J, Panneerselvam J, Srivastava A, Kim TD, Janknecht R, Herman T, Ramesh R, Munshi A (2017). YAP1 inhibition radiosensitizes triple negative breast cancer cells by targeting the DNA damage response and cell survival pathways. Oncotarget.

[31] Zhao Y, Adjei AA (2014). The clinical development of MEK inhibitors. Nat Rev Clin Oncol.

[32] Loh CY, Arya A, Naema AF, Wong WF, Sethi G, Looi CY (2019). Signal transducer and activator of transcription (STATs) proteins in cancer and inflammation: functions and therapeutic implication. Front Oncol.

[33] Liu F, Zhang H, Song H (2017). Upregulation of MEK5 by Stat3 promotes breast cancer cell invasion and metastasis. Oncol Rep.

[34] Summy JM, Gallick GE (2003). Src family kinases in tumor progression and metastasis. Cancer Metastasis Rev.

[35] Choi SK, Kim HS, Jin T, Hwang EH, Jung M, Moon WK (2016). Overexpression of the miR-141/200c cluster promotes the migratory and invasive ability of triple-negative breast cancer cells through the activation of the FAK and PI3K/AKT signaling pathways by secreting VEGF-A. BMC Cancer.

[36] McCubrey JA, Steelman LS, Bertrand FE, Davis NM, Sokolosky M, Abrams SL, Montalto G, D'Assoro AB, Libra M, Nicoletti F (2014). GSK-3 as potential target for therapeutic intervention in cancer. Oncotarget.

[37] Bachelder RE, Yoon SO, Franci C, de Herreros AG, Mercurio AM (2005). Glycogen synthase kinase-3 is an endogenous inhibitor of snail transcription: implications for the epithelial-mesenchymal transition. J Cell Biol.

[38] Cross DA, Alessi DR, Cohen P, Andjelkovich M, Hemmings BA (1995). Inhibition of glycogen synthase kinase-3 by insulin mediated by protein kinase B. Nature.

[39] Domoto T, Pyko IV, Furuta T, Miyashita K, Uehara M, Shimasaki T, Nakada M, Minamoto T (2016). Glycogen synthase kinase-3 is a pivotal mediator of cancer invasion and resistance to therapy. Cancer Sci.

[40] Kazi A, Xiang S, Yang H, Delitto D, Trevino J, Jiang RHY, Ayaz M, Lawrence HR, Kennedy P, Sebti SM (2018). GSK3 suppression upregulates beta-catenin and c-Myc to abrogate KRas-dependent tumors. Nat Commun.

[41] Nayak D, Kumar A, Chakraborty S, Rasool RU, Amin H, Katoch A, Gopinath V, Mahajan V, Zilla MK, Rah B (2017). Inhibition of Twist1-mediated invasion by Chk2 promotes premature senescence in p53-defective cancer cells. Cell Death Differ.

[42] Adorno M, Cordenonsi M, Montagner M, Dupont S, Wong C, Hann B, Solari A, Bobisse S, Rondina MB, Guzzardo V (2009). A mutant-p53/Smad complex opposes p63 to empower TGF beta-induced metastasis. Cell.

[43] Muller PAJ, Caswell PT, Doyle B, Iwanicki MP, Tan EH, Karim S, Lukashchuk N, Gillespie DA, Ludwig RL, Gosselin P (2009). Mutant p53 drives invasion by promoting integrin recycling. Cell.

[44] Song H, Hollstein M, Xu Y (2007). P53 gain-of-function cancer mutants induce genetic instability by inactivating ATM. Nat Cell Biol.

[45] Girardini JE, Napoli M, Piazza S, Rustighi A, Marotta C, Radaelli E, Capaci V, Jordan L, Quinlan P, Thompson A (2011). A Pin1/mutant p53 Axis promotes aggressiveness in breast Cancer. Cancer Cell.

[46] Kao J, Salari K, Bocanegra M, Choi YL, Girard L, Gandhi J, Kwei KA, Hernandez-Boussard T, Wang P, Gazdar AF (2009). Molecular profiling of breast cancer cell lines defines relevant tumor models and provides a resource for cancer gene discovery. PLoS One.

[47] Ebbesen SH, Scaltriti M, Bialucha CU, Morse N, Kastenhuber ER, Wen HY, Dow LE, Baselga J, Lowe SW (2016). Pten loss promotes MAPK pathway dependency in HER2/neu breast carcinomas. Proc Natl Acad Sci U S A.

[48] Shah SP, Roth A, Goya R, Oloumi A, Ha G, Zhao Y, Turashvili G, Ding J, Tse K, Haffari G (2012). The clonal and mutational evolution spectrum of primary triple-negative breast cancers. Nature.

[49] Castellano E, Downward J (2011). RAS interaction with PI3K: more than just another effector pathway. Genes Cancer.

[50] Sabbagh A, Pasmant E, Imbard A, Luscan A, Soares M, Blanche H, Laurendeau I, Ferkal S, Vidaud M, Pinson S (2013). NF1 molecular characterization and neurofibromatosis type I genotype-phenotype correlation: the French experience. Hum Mutat.

[51] Xiang T, Ohashi A, Huang Y, Pandita TK, Ludwig T, Powell SN, Yang Q (2008). Negative regulation of AKT activation by BRCA1. Cancer Res.

[52] Wang C, Bai F, Zhang LH, Scott A, Li E, Pei XH (2018). Estrogen promotes estrogen receptor negative BRCA1-deficient tumor initiation and progression. Breast Cancer Res.

[53] Coene ED, Gadelha C, White N, Malhas A, Thomas B, Shaw M, Vaux DJ (2011). A novel role for BRCA1 in regulating breast cancer cell spreading and motility. J Cell Biol.

[54] Wilson LJ, Linley A, Hammond DE, Hood FE, Coulson JM, MacEwan DJ, Ross SJ, Slupsky JR, Smith PD, Eyers PA (2018). New perspectives, opportunities, and challenges in exploring the human protein kinome. Cancer Res.

