# Supplementary material for: Phytochemicals inhibit migration of triple negative breast cancer cells by targeting kinase signaling
Source: BMC Cancer. 2020 Jan 2;20:4. doi: 10.1186/s12885-019-6479-2 (PMC6941316; doi:10.1186/s12885-019-6479-2)
Supplement: Supplementary file 1 — Additional file 1: Table S1. Coordinates of protein kinases in the phospho-kinase dot blot array. Figure S1. (a) Migration of TNBC cells into the cell-excluded gap after 48 hrs without any treatment. (b) Subtypes of TNBC cell lines and their major mutations. Figure S2. Basal-like HCC1937 cells migrated into and occupied the gap almost within 24 hrs. Scale bar is 1 mm. Figure S3. Cytotoxicity analysis TNBC cells treated with (a) fisetin and (b) quercetin. Each data point represents the mean of 8 samples and error bars represent standard error from the mean. (c-e) Representative images of Hs578T show that the spindle-like mesenchymal morphology of cells changes to epithelial cell morphology with fisetin and quercetin treatments. Note that with fisetin treatment of HCC1806 cells and quercetin treatment of HCC1937 cells, a sigmoidal curve could not be fitted to the dose response data. Figure S4. Baseline relative phosphorylation of 43 protein kinases and 2 related signaling proteins in nine TNBC cell lines without any treatments. The hierarchical clustering identified 4 major clusters: Cluster 1 is highlighted with a red box (high baseline activity), Cluster 2 is highlighted with an orange box (high to moderate baseline activity), Cluster 3 is highlighted with a green box (moderate baseline activity), and Cluster 4 is highlighted with blue box (low baseline activity). Figure S5. Fisetin and quercetin treatments are non-cytotoxic to TNBC cells. Viability of nine TNBC cell lines after treatments with (a) 200 µM fisetin and (b) 200 µM quercetin for 6 hours. Figure S6. GSK1059615 treatment dose-dependently downregulated p-WNK1. (a-d) Western blots of p-WNK1 and t-WNK in TNBC cells treated with GSK1059615 for 6 hrs. Figure S7. Combination treatment of TNBC cells with GSK1059615 and WNK463 inhibitors produced an additive effect, suggesting that p-WNK1 is a p-AKT effector. (a) Western blot for single agent and combination treatments for 6 hrs. (b-c) Levels of p-AKT/t-AKT and p [file 12885_2019_6479_MOESM1_ESM.pdf]

Supplementary Information

Supplementary Table S1. Coordinates of protein kinases in the phospho-kinase dot blot array.

Membrane/Coordinate	Target/Control
A-A1, A2	Reference spot
A-A3, A4	p38alpha
A-A5, A6	ERK1/2
A-A7, A8	JNK1/2/3
A-A9,A10	GSK-3alpha/beta
B-A13, A14	p53
B-A17, A18	Reference spot
A-B3, B4	EGFR
A-B5, B6	MSK1/2
A-B7, B8	AMPK alpha1
A-B9, B10	Akt1/2/3
B-B11,B12	Akt1/2/3
B-B13,B14	p53
A-C1,C2	TOR
A-C3,C4	CREB
A-C5,C6	HSP27
A-C7,C8	AMPK alpha2
A-C9,C10	beta-catenin
B-C11,C12	p70 S6 Kinase
B-C13,C14	p53
B-C15,C16	c-Jun
A-D1,D2	Src
A-D3,D4	Lyn
A-D5,D6	Lck
A-D7,D8	STAT2
A-D9,D10	STAT5a
B-D11,D-12	p70 S6 Kinase
B-D13,D14	RSK1/2/3
B-D15,D16	Enos
A-E1, E2	Fyn
A-E3,E4	Yes
A-E5, E6	Fgr
A-E7, E8	STAT6
A-E9, E-10	STAT5b
B-E11,E12	STAT3
B-E13,E14	p27
B-E15, E16	PLC-ÿ1
A-F1,F2	Hck
A-F3,F4	Chk-2
A-F5,F6	FAK
A-F7, F8	PDGFRβ
A-F9, F10	STAT5a/b
B-F11, F12	STAT3
B-F13, F14	WNK1
B-F15, F16	PYK2
B-G1,G2	Reference spot
B-G3,G4	PRAS40
A-G9,G10	PBS(Negative Control)
B-G11, G12	HSP60
B-G17,G18	PBS(Negative Control)

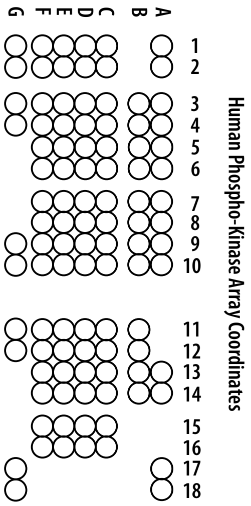

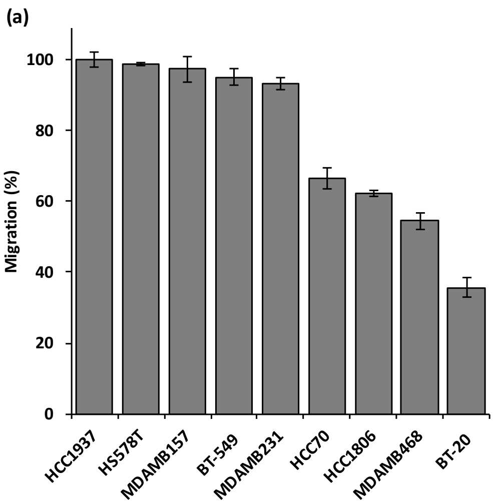

(b)

TNBC subtype	Cell line	Mutations	Ethnicity
Basal Like			
BL1	HCC1937	BRCA1; TP53; MAPK13;MDC1	Caucasian
BL1	MDA-MB-468	PTEN;RB1;SMAD4;TP53	African-American
BL2	HCC70	PTEN; TP53	African-American
BL2	HCC1806	CDKN2A;TP53;UTX	African-American
Mesenchymal like			
M	BT-549	PTEN; RB1; TP53	Caucasian
MSL	HS578T	CDKN2A; HRAS; TP53	Caucasian
MSL	MDA-MB-157	NF1; TP53	African-American
MSL	MDA-MB-231	BRAF; CDKN2A;KRAS;NF2;TP53;PDGFRA	Caucasian
Unclassified			
-	BT20	CDKN2A; PIK3CA; TP53	Caucasian

Source: J. Kao et al., PLoS One 4 (2009) e6146

Supplementary Figure S1. (a) Migration of TNBC cells into the cell-excluded gap after 48 hrs without any treatment. (b) Subtypes of TNBC cell lines and their major mutations.

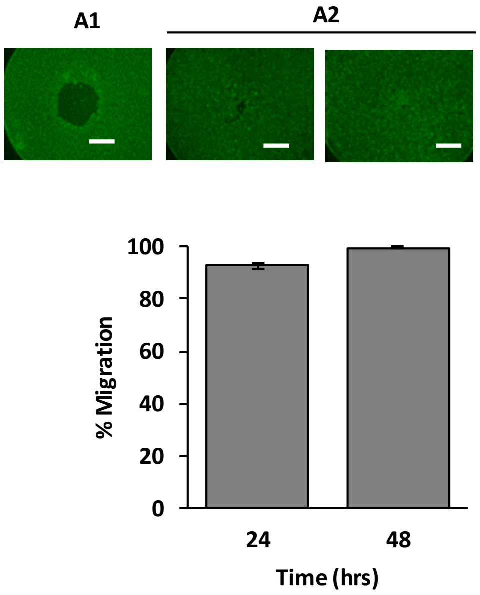

Supplementary Figure S2. Basal-like HCC1937 cells migrated into and occupied the gap almost within 24 hrs. Scale bar is 1 mm.

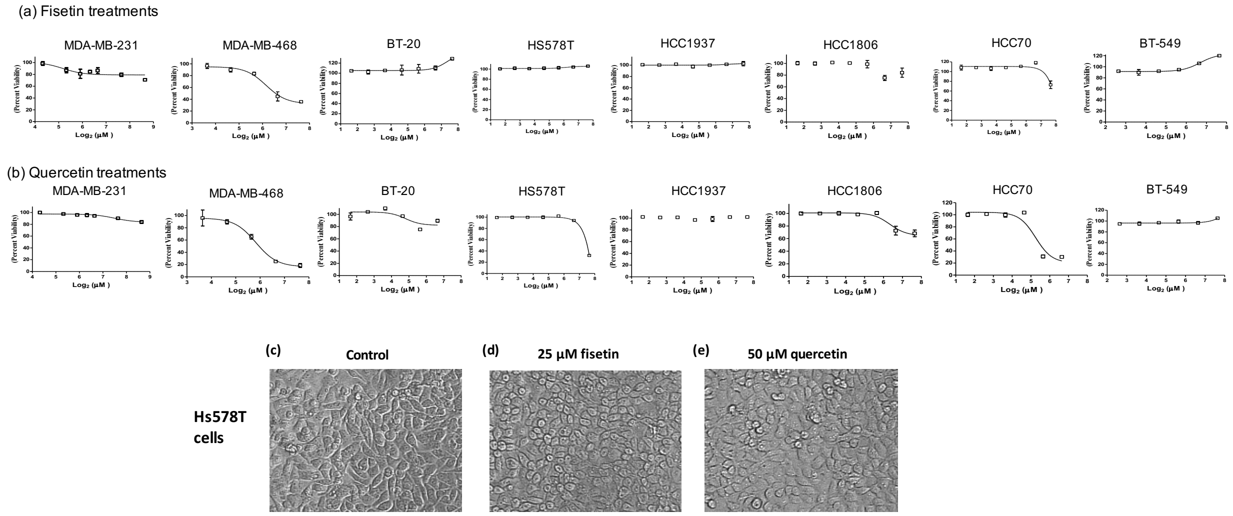

Supplementary Figure S3. Cytotoxicity analysis TNBC cells treated with (a) fisetin and (b) quercetin. Each data point represents the mean of 8 samples and error bars represent standard error from the mean. (c-e) Representative images of Hs578T show that the spindle-like mesenchymal morphology of cells changes to epithelial cell morphology with fisetin and quercetin treatments. Note that with fisetin treatment of HCC1806 cells and quercetin treatment of HCC1937 cells, a sigmoidal curve could not be fitted to the dose response data.

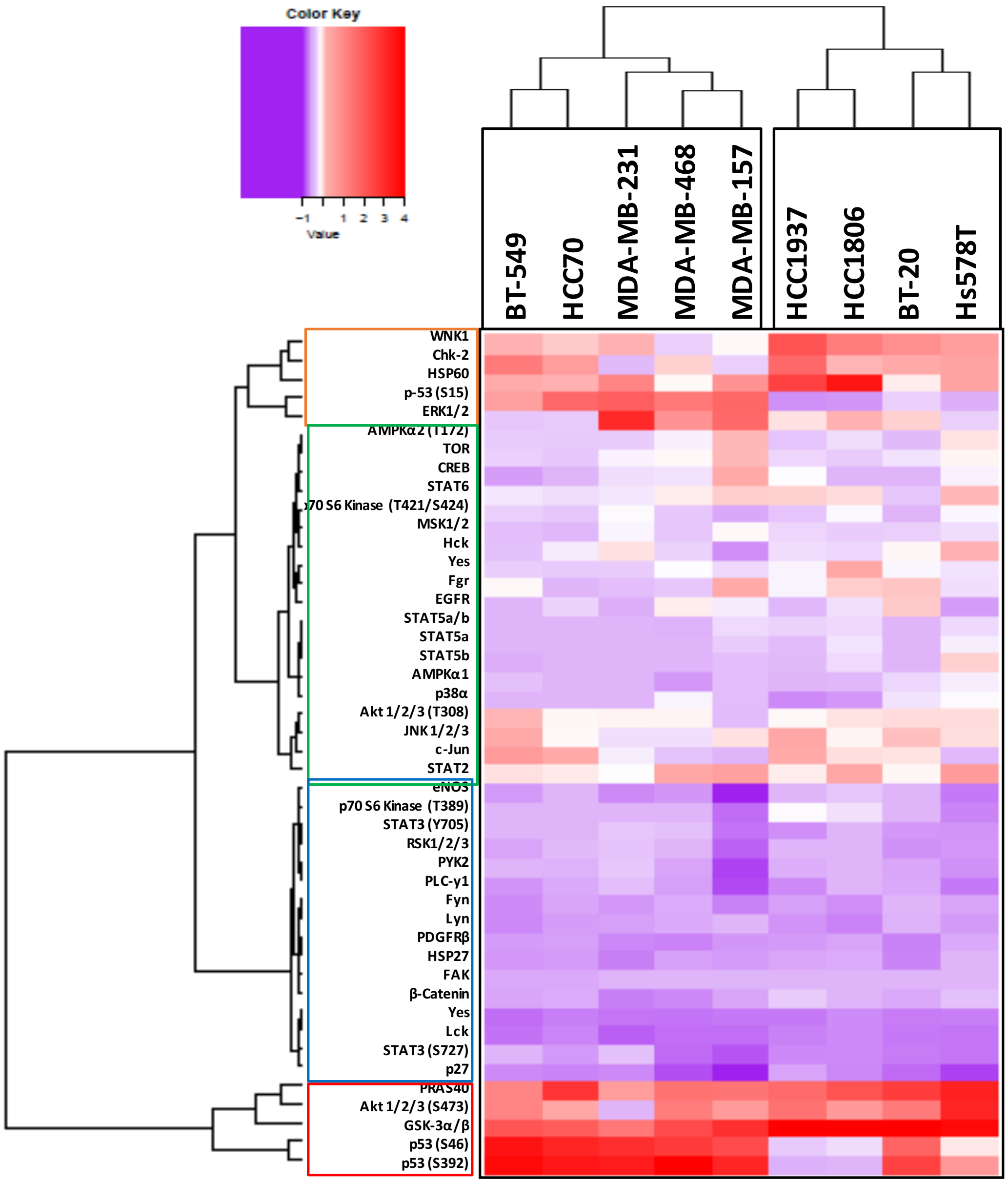

Supplementary Figure S4. Baseline relative phosphorylation of 43 protein kinases and 2 related signaling proteins in nine TNBC cell lines without any treatments. The hierarchical clustering identified 4 major clusters: Cluster 1 is highlighted with a red box (high baseline activity), Cluster 2 is highlighted with an orange box (high to moderate baseline activity), Cluster 3 is highlighted with a green box (moderate baseline activity), and Cluster 4 is highlighted with blue box (low baseline activity).

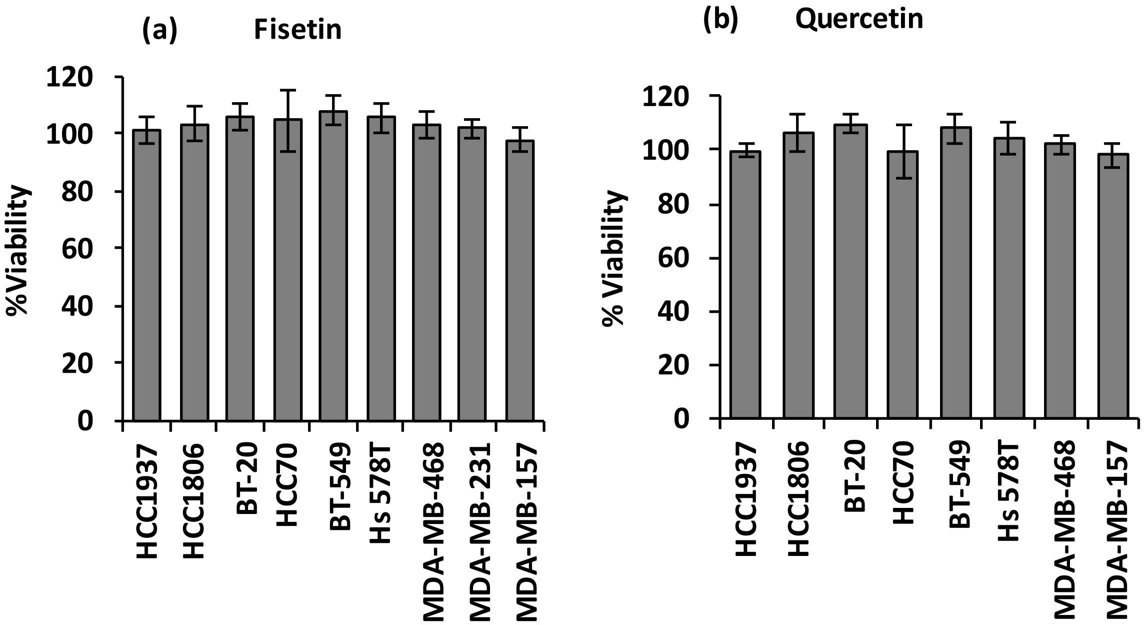

Supplementary Figure S5. Fisetin and quercetin treatments are non-cytotoxic to TNBC cells. Viability of nine TNBC cell lines after treatments with (a) 200 μM fisetin and (b) 200 μM quercetin for 6 hours.

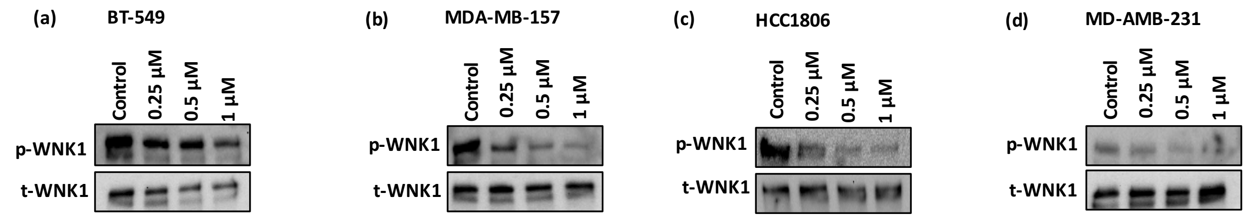

Supplementary Figure S6. GSK1059615 treatment dose-dependently downregulated p-WNK1. (a-d) Western blots of p-WNK1 and t-WNK in TNBC cells treated with GSK1059615 for 6 hrs.

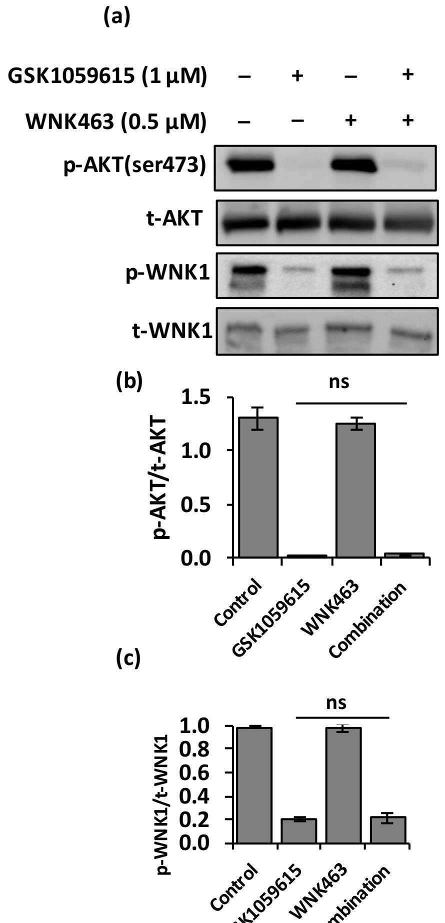

Supplementary Figure S7. Combination treatment of TNBC cells with GSK1059615 and WNK463 inhibitors produced an additive effect, suggesting that p-WNK1 is a p-AKT effector. (a) Western blot for single agent and combination treatments for 6 hrs. (b-c) Levels of p-AKT/t-AKT and p-WNK1/t-WNK1 in HCC1806 cells, respectively. ns represents lack of statistically significant difference.

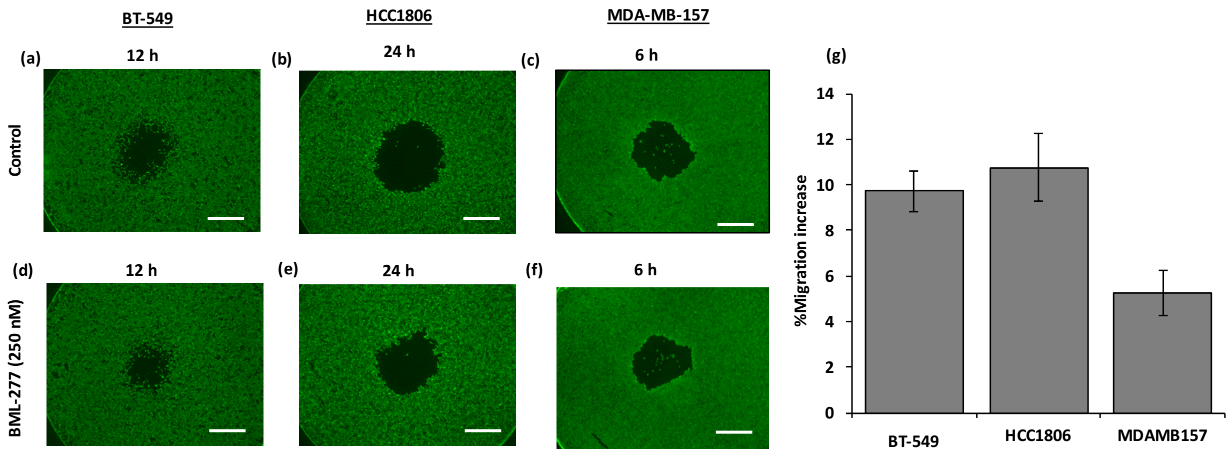

Supplementary Figure S8. CHK2 inhibition promoted migration of different TNBC cells. Images of cell migration (a-c) without any treatment and (d-f) treatments with BML-277. Scale bar is 1 mm. (g) Quantified increased migration of TNBC cells by CHK2 inhibition. Each bar represents a mean of 8 samples, and error bars represent standard error from mean.
